# Variation in mitochondrial DNA affects locomotor activity and sleep in *Drosophila melanogaster*

**DOI:** 10.1038/s41437-022-00554-w

**Published:** 2022-06-28

**Authors:** Lucy Anderson, M. Florencia Camus, Katy M. Monteith, Tiina S. Salminen, Pedro F. Vale

**Affiliations:** 1 Institute of Ecology and Evolution, School of Biological Sciences, University of Edinburgh, Edinburgh, UK; 2 Research Department of Genetics, Evolution and Environment, University College London, Gower Street, London, WC1E 6BT UK; 3 Faculty of Medicine and Health Technology, Tampere University, Tampere, Finland

**Keywords:** Behavioural genetics, Drosophila, Genetic models

## Abstract

Mitochondria are organelles that produce cellular energy in the form of ATP through oxidative phosphorylation, and this primary function is conserved among many taxa. Locomotion is a trait that is highly reliant on metabolic function and expected to be greatly affected by disruptions to mitochondrial performance. To this end, we aimed to examine how activity and sleep vary between *Drosophila melanogaster* strains with different geographic origins, how these patterns are affected by mitochondrial DNA (mtDNA) variation, and how breaking up co-evolved mito-nuclear gene combinations affect the studied activity traits. Our results demonstrate that *Drosophila* strains from different locations differ in sleep and activity, and that females are generally more active than males. By comparing activity and sleep of mtDNA variants introgressed onto a common nuclear background in cytoplasmic hybrid (cybrid) strains, we were able to quantify the among-line variance attributable to mitochondrial DNA, and we establish that mtDNA variation affects both activity and sleep, in a sex-specific manner. Altogether our study highlights the important role that mitochondrial genome variation plays on organismal physiology and behaviour.

## Introduction

Mitochondria are key organelles in a range of critical metabolic processes and are the primary energy producers for the eukaryotic cell. In addition to this primary role, they are also involved in a range of other vital processes that control key aspects of cellular growth and regulation such as signalling (Chandel Navdeep 2015), cellular differentiation (Vega-Naredo et al. 2014), cell death (Wang and Youle 2009) and immunity (Buchanan et al. 2018; Salminen and Vale 2020). The mitochondrial machinery responsible for ATP production via oxidative phosphorylation (OXPHOS) is jointly encoded by the mitochondrial (mtDNA) and nuclear genomes. While the mtDNA genome encodes for 13 protein-coding OXPHOS genes, the nuclear genome encodes the majority of the OXPHOS subunits as well as over 1200 other genes required for mitochondrial function (Anderson et al. 1981; Gray et al. 1999a; Gray et al. 1999b; Lang et al. 1999). In addition to vast differences in genome size, there are large differences in copy number between the genomes, with up to hundreds of mtDNA copies inhabiting each diploid cell (Robin and Wong 1988). Consequently, precise and synchronised coordination between the two genomes is required for proper assembly and function of the components of the electron transport chain and mitochondrial functions. Disruptions to this system—by mutations in the mitochondrial or nuclear counterparts—can have consequences on a wide range of life-history phenotypes (Hill et al. 2019), and in severe cases lead to mitochondrial disease (DiMauro and Schon 2003; Salminen et al. 2019; Schon et al. 2012; Smeitink et al. 2001; Wallace 1994). A consequence of this tight intergenomic partnership is that any trait with heavy metabolic underpinnings is reliant on the compatibility between mitochondrial and nuclear genomes.

Mitochondrial dysfunction affects a wide range of metabolic and behavioural traits, given the key role of mitochondria in energetics (Ghaoui and Sue 2018). Two such traits which are vital for everyday function are locomotor activity and sleep. Sleep is integral to regular brain function, influencing processes such as learning and memory (Ganguly-Fitzgerald et al. 2006), and plays a role in cellular processes such as metabolic recovery and oxidative stress (Trivedi et al. 2017). Continued sleep deprivation results in fatality for both invertebrate and vertebrate species (Potdar and Sheeba 2013; Rechtschaffen et al. 1989; Shaw et al. 2002). In humans, sleep deprivation is associated with an increased risk of metabolic and cognitive disorders (Harbison et al. 2013). In *Drosophila*, sleep deprivation has been shown to be connected to mitochondrial bioenergetics and causes mitochondrial dysfunction (Rodrigues et al. 2018). However, investigation of sleep as a consequence of mitochondrial dysfunction appears to be an understudied aspect of mitochondrial disease (Brunetti et al. 2021). It is also unclear how naturally occurring mtDNA variation may affect sleep.

Locomotion is also heavily reliant on metabolic function, with studies finding a strong positive correlation between activity and resting metabolic rate (Videlier et al. 2019). Moreover, both increased fatigue and exercise intolerance have been linked to several metabolic disorders originating from mitochondrial dysfunction (Filler et al. 2014; Sujkowski et al. 2019). For instance, *Drosophila* models of neuromuscular degeneration have shown a progressive decrease in fly activity with time (Bar et al. 2018). Additionally, nutritional metabolic interventions in the form of dietary restriction have shown to change activity patterns (Ghimire and Kim 2015), while physiologically costly immune stimulation has also been found to result in reduced locomotor activity in some insect species (Vale and Jardine 2015; Gupta et al. 2017; Vale et al. 2018; Vincent et al. 2021).

Given their tight link with metabolism, mutations affecting mitochondrial function are predicted to affect both sleep and locomotor activity. A previous review of primary mitochondrial diseases described sleep disorders in humans associated with mutations in mitochondrial DNA (Ramezani and Stacpoole 2014), but in general, we know little about how mitochondrial variation may affect activity and sleep patterns (Fogle et al. 2019; Ramezani and Stacpoole 2014). The fruit fly, *Drosophila melanogaster*, offers a powerful system to address the link between mitochondrial variation and activity and sleep disruption. *Drosophila* is an established genetic model system, including the study of mito-nuclear effects on various phenotypic traits (Camus and Dowling 2018; Hoekstra et al. 2013; Holmbeck et al. 2015; Zhu et al. 2014). As mtDNA is maternally inherited, introgression enables the generation of flies with specific combinations of nDNA and mtDNA. Generation of cytoplasmic hybrids (cybrids) therefore allows the effects of mtDNA mutations to be disentangled from nuclear genome variation (Rand et al. 2004). A large body of work using *Drosophila* cybrid lines has established that several measures of life-history phenotypes are modulated by changes in the mitochondrial genome, including aging (Camus et al. 2012; Camus et al. 2020; Rand et al. 2006), fitness (Camus and Dowling 2018; Mossman et al. 2019; Salminen et al. 2017), and metabolic rate (Nagarajan-Radha et al. 2020).

*Drosophila* is also an established model for the study of sleep and circadian rhythms and displays a state of quiescence that shares critical features of mammalian sleep (Shaw et al. 2000). These similarities include an elevated arousal threshold (Shaw et al. 2000), altered brain electrical activity (Nitz et al. 2002) and a decrease in amount of sleep as flies age (Hendricks et al. 2000). Furthermore, gene expression associated with ‘waking’ in fruit flies has been shown to correlate with ‘waking’ genes in mammals (Shaw et al. 2000). A relevant example is the mtDNA-encoded Cytochrome oxidase C, subunit I, which has been demonstrated to have elevated expression during the initial hours following sleep in both *Drosophila* and rats (Shaw et al. 2000). This homology between *Drosophila* and mammalian sleep, combined with the knowledge that many of the genetic and molecular regulators of sleep are conserved between flies and humans (Crocker and Sehgal 2010), has prompted extensive use of the fruit fly as a genetically tractable model organism in the study of sleep.

To specifically address the role of variation in the mitochondrial DNA on activity and sleep, we examined the sleep–wake cycles and activity profiles of a worldwide collection of eight *D. melanogaster* lines, in addition to a set of derived cybrid lines which contained each of the eight mtDNA variants introgressed onto a single common nuclear background. This experimental setup allowed us to investigate the baseline activity and sleep profiles of each line and to assess the contribution of mtDNA to these phenotypes. Further, because the mitochondrial genome of each line presents unique mtDNA variation at the haplotype level as well as common variants at the haplogroup level (Salminen et al. 2017) we were able to test if variation in sleep and activity patterns were associated with specific haplotypes.

## Materials and methods

### Fly strains, backcrossing and rearing conditions

We sourced eight wild-type *D. melanogaster* strains, with distinct geographic origins (Table 1), originally obtained from the Drosophila Stock Center (Bloomington, IN). Based on the mtDNA coding region variation the eight mtDNA variants form two distinct haplogroups with a set of few common replacement variants present in haplogroup I in OXPHOS complexes I (*ND1*; V190M, *ND2*; I277L, *ND5*; M502I) and V (*ATP6*; S538P and M559V) (Salminen et al. 2017). Haplogroup II contains European mtDNA variants (mtPYR2, mtLS and mtBS1), whereas haplogroup I contains variants from different continents (mtORT, mtKSA2, mtBOG1, mtWT5A and mtM2) (Table 1). Cybrid lines were created earlier by backcrossing females from each strain (carrying unique mtDNA variant) to males from the nuclear-donor strain of the Oregon RT strain (Oregon R strain maintained long-term in Tampere, Finland; ORT) for at least 12 generations (Salminen et al. 2017). This resulted in a total of 16 strains; 8 of which were the Bloomington-derived strains representing coevolved mito-nuclear combinations and 8 were nORT mtDNA*x* cybrids. All lines were cultured on standard Lewis medium (Lewis 1960), supplemented with yeast, under 12: 12 light: dark cycles at 25 °C and 60% humidity. Flies were propagated by placing 30, 2–4-day old females and males on food vials for 3–4 days, with adults being discarded and egg clutches kept. This rearing regime maintained egg densities low enough to prevent larval overcrowding.

**Table 1:** *Drosophila melanogaster* strains used in the study.

Strain	mtDNA haplogroup	GenBank accession number	Geographic origin	Latitude	Amino acid replacement (OXPHOS complex, gene)
ORT	I	KY559383	–	–	–
KSA2	I	KY559384	Kariba Dam, Zimbabwe	17°S	D_21_N (III, *CYTB*) A_75_T (IV, *COIII*)
BOG1	I	KY559386	Bogota, Colombia	4°N	A_356_T (I, *ND5*)
WT5A	I	KY559387	Red Top Mountain, GA, USA	34°N	–
M2	I	KY559388	Australia	25°S	M_280_V (I, *ND2*) V_161_L (I, *ND4*) A_106_T (IV, *COI*) M_185_I (V, *ATP6*)
PYR2	II	KY559389	Pyrenees, Spain	43°N	N_114_D (I, *ND6*) V_264_M (III, *CYTB*)
LS	II	KY559390	Lausanne, Switzerland	46°N	S_59_F (I, *ND5*) D_13_N (IV, *COIII*)
BS1	II	KY559391	Barcelona, Spain	41°N	G_58_S (IV, *COII*)

Strains were obtained from Bloomington stock centre and originally collected from different continents. GenBank accession numbers refer to the mtDNA coding region sequences. Haplotype-specific amino acid replacements have been added here based on Salminen et al. (2017).

### Experimental design

The Drosophila Activity Monitor (DAM2, Trikinetics) was used to collect all experimental data on fly activity and sleep cycles (Chiu et al. 2010). Prior to the experiment, a solution of 8% sucrose: 2% agar was prepared in distilled water and autoclaved for sterilisation and stored at room temperature before re-melting for use. DAM2 tubes (5 mm diameter, 65 mm length) were prepared by adding ~1 cm sucrose-agar medium to one end of the tube and sealing with a rubber cap. This provided a source of food and moisture for the fly during the experiment. Flies from each coevolved and cybrid strain were collected upon eclosion and kept in food vials for 2 days. Flies were anesthetised with CO_2_, sorted by sex and transferred into a DAM2 tube using a fine paintbrush. After the anesthetised fly had been inserted into the DAM2 tube it was closed with a rubber cap containing a small hole for ventilation (Chiu et al. 2010).

Fifteen individuals per sex and strain were tested across five experimental blocks, with 3–5 replicates per block for each line/sex combination positioned haphazardly in the activity monitors. Each monitor had a null or blank control in which a recorded position contained an empty tube or no tube, respectively. Monitoring of fly activity and sleep lasted for three continuous days. Each DAM2 tube is bisected by an infra-red beam and locomotor activity movement was recorded whenever a fly broke the beam. The number of activity counts (beam breaks) generated by the DAM was used to quantify locomotor activity. Sleep was defined as a 5 min time-bin with no registered activity (Andretic and Shaw 2005). To determine whether individuals/flies are more active simply because they sleep less, we calculated the proportion of time each replicate spends sleeping, as well as the mean activity count during awake periods. The experiments were run under 12:12 light:dark cycles at 25 °C at constant temperature and humidity.

### Statistical analysis

Total activity count, mean awake activity and proportion of time sleeping were calculated for each individual fly (see also Vale and Jardine 2015). Four flies (out of 480) died during the experiment and were excluded from analysis. We analysed the co-evolved and cybrid lines separately, using otherwise identical statistical models. Total activity count and mean awake activity were analysed using linear mixed-effects models (LME). Models fitted ‘line’, ‘sex’ and their interaction as categorical fixed effects. Variation in proportion of time asleep was analysed using generalised linear effects models (GLME) assuming binomial distributed error, which also fitted ‘line’, ‘sex’ and their interaction as categorical fixed effects. All models included the random effect of ‘replicate’ nested within ‘block’ to account for variation between individuals among different blocks. We also investigated if breaking up co-evolved mito-nuclear gene complexes affected each of these behavioural outputs. This analysis therefore included the coevolved and cybrid fly lines to analyse the effect of mtDNA variant on its coevolved or cybrid nuclear background. Similar model structure was used as described above for each response variable, where each model fitted ‘type’ (coevolved or cybrid), ‘sex’ and their interactions as categorical fixed effects, and ‘line’ as a random effect nested within type. R version 1.1.4 (Team RC 2019) was used for analysis and plots, using packages *ggplot2* (Wickham 2009), *dplyr* (Wickham et al. 2019), lme4 (Bates et al. 2012), car (Fox and Weisberg 2011) and plotrix (Lemon 2016). All datasets and full R code for all analyses can be found at 10.5281/zenodo.5573904.

## Results

### Coevolved fly lines show sex-specific natural variation in sleep and activity patterns

**Fig. 1:**
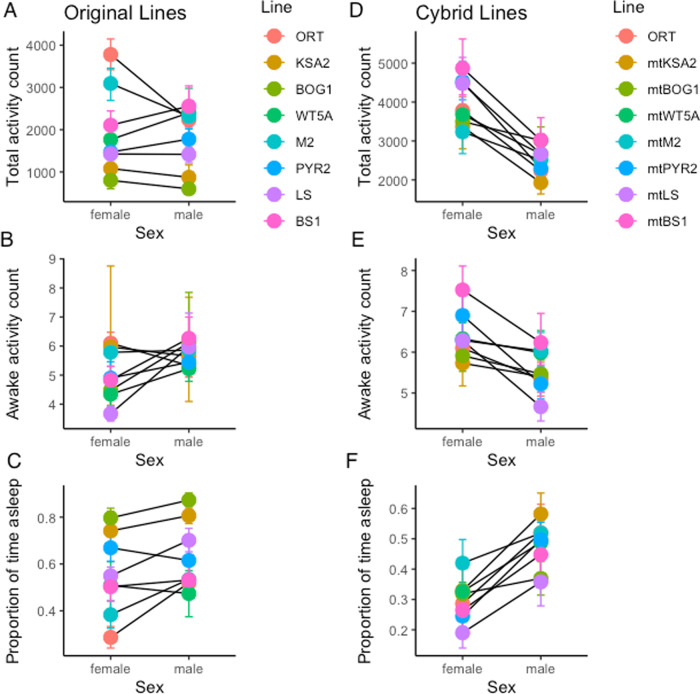
Locomotor activity and sleep in females and males of the coevolved and cybrid lines. The total number of activity events recorded over three days in original (**A**) and cybrid (**D**) lines. The total number of activity events recorded when the flies were awake in original (**B**) and cybrid (**E**) lines. The proportion of time that flies were asleep, defined as 5 min of inactivity in original (**C**) and cybrid (**F**) lines. See Figs S1-S4 for individual actograms and boxplots for each line. See Table 1 for details of each line. See Table 2 for outputs of statistical models and Table S1 for random effect variance components.

We first evaluated the sleep and activity patterns of genetically and geographically diverse fly lines carrying natural, and presumably coevolved, mito-nuclear combinations (Table 1). Activity profiles showed that all lines were crepuscular, exhibiting a peak of activity at the onset of the dark period (Fig. S1). In the majority of strains, females were significantly more active than males (14% more active—female mean total activity: 1988, male: 1750). The extent of this difference was influenced by genotype and in lines BS1 and WT5A, males were more active than females (Table 2 ‘Line × Sex’ effect; Fig. 1A and Fig. S2). Overall, males spent a higher proportion of time asleep (64% of the time) compared to females (55% of the time), and the extent of this difference varied between genetic backgrounds (Table 2 ‘Line × Sex’ effect). Notably, while males slept for a greater proportion of the day, they were slightly more active while awake (5.74 recorded movements per 5-min bout) compared to females (5 recorded movements per 5-min bout), and again the extent of this variation differed between lines (Table 2 ‘Line × Sex’ effect; Fig. 1B, C). This suggests that although females exhibit higher total activity, this is due to females spending more time awake (sleeping less) rather than having higher levels of activity when awake.

**Table 2:** Summaries of test statistics for fixed effects in linear models.

	Total Activity Count		Proportion of time asleep		Mean awake activity	
	*χ*^2^	*p*	*χ*^2^	*p*	*χ*^2^	*p*
Original Lines						
Line	320.74	<0.001	12808.10	<0.001	10.56	0.159
Sex	3.79	0.052	1063.30	<0.001	9.40	0.002
Line × Sex	59.90	<0.001	1943.80	<0.001	16.48	0.021
Cybrid lines						
Line	40.80	<0.001	2563.42	<0.001	47.32	<0.001
Sex	134.56	<0.001	5683.25	<0.001	45.47	<0.001
Line × Sex	21.95	0.003	904.27	<0.001	20.11	0.005
Coevolved vs. Novel						
Type	18.62	<0.001	10.42	0.0012	8.22	<0.001
Sex	75.59	<0.001	4985.67	<0.001	0.001	0.975
Type × Sex	50.99	<0.001	720.34	<0.001	27.70	<0.001

See methods for model details.

### Mitochondrial genome variation affects activity and sleep patterns of the cybrid lines

By introgressing the eight mtDNA variants onto a common nuclear background (ORT), we were able to evaluate how much of the variance in locomotor activity was affected by the mtDNA and newly created mito-nuclear combinations (Figs. S3, S4, Table S1). In general, comparing total activity counts showed that as observed with coevolved lines, females of each cybrid line were significantly more active than cybrid males (45% more active - female mean total activity: 3847, male: 2652), and the extent of this variation was mtDNA specific (Table 2 ‘Line × Sex’ effect; Fig. 1E and Fig. S4). Therefore, compared to the coevolved lines, cybrid lines exhibited a substantial 75% increase in the total activity on average (3266 recorded movements compared to 1867 on average in the original lines), and the differences between sexes, while consistent in showing females are more active, were also more pronounced in cybrid lines (45% compared to 14%) (Fig. 1A, D). Males were found to sleep for a significantly larger proportion of time (46% of the time) compared to females (30% of the time) (Fig. 1B, E), although unlike the co-evolved lines, the female cybrids were generally more active when awake (6.3 recorded movements per 5-min bout) when compared to males (5.6 recorded movements per 5-min bout) (Fig. 1C, F). Both of these differences were also mediated by mtDNA variation (Table 2 ‘Line × Sex’ effect). Therefore, when the effects of individual mitochondrial genomes are isolated on a common nuclear background females are both more active while awake and also spend less time sleeping than males.

### Haplogroup-specific mtDNA variation can be seen in the activities of cybrid females

**Fig. 2:**
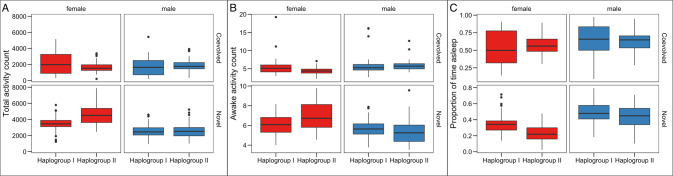
Haplogroup effect. Locomotor activities and sleep in coevolved and cybrid females and males based on the mtDNA haplogroup division. **A** Total activity counts over three days. **B** Activity of the flies when awake. **C** The proportion of time the flies spent sleeping.

Based on the mtDNA coding region variation the eight mtDNA variants form two distinct haplogroups with a set of few common replacement variants present in haplogroup I in OXPHOS complexes I (*ND1*; V190M, *ND2*; I277L, *ND5*; M502I) and V (*ATP6*; S538P and M559V) (Salminen et al. 2017). Haplogroup I contains the haplotypes mtORT, mtKSA2, mtBOG1, mtWT5A and mtM2, as the haplogroup II contains the haplotypes mtPYR2, mtLS and mtBS1 (Table 1). Haplogroup division did not cause clear differences in the sleep-wake activities when the haplogroup-specific mtDNA variants were present in their coevolved nuclear backgrounds and the sex differences that were observed earlier with the coevolved lines were decreased (Table 3, Fig. 2. upper row). However, when the haplogroup I and II mtDNA variants were placed on a novel common nuclear background in the cybrid lines, we observed that haplogroup II females we more active than haplogroup I females (Table 3, Fig. 2, lower row A). Haplogroup II females were also more active when awake and slept for a smaller proportion of time than haplogroup I females (Table 3, Fig. 2 lower rows B and C).

**Table 3:** Summaries of test statistics for fixed effects in testing the effect of haplogroup and sex on activity and sleep in coevolved and cybrid lines.

Haplogroup effect	Total Activity		Mean Awake activity		Proportion time asleep	
	*χ*^2^	*p*	*χ*^2^	*p*	*χ*^2^	*p*
Coevolved Lines						
Haplogroup	0.78	0.378	1.37	0.24	1.66	0.197
Sex	3.07	0.08	8.71	0.003	30.39	<0.001
Haplogroup × Sex	7.77	0.05	4.66	0.03	0.70	0.404
Cybrid lines						
Haplogroup	25.09	<0.001	3.99	0.05	18.00	<0.001
Sex	123.46	<0.001	37.54	<0.001	61.78	<0.001
Haplogroup × Sex	12.74	0.0003	15.53	<0.001	0.16	0.69

**Fig. 3:**
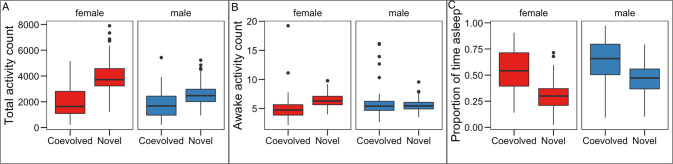
A comparison of Coevolved and Novel Mito-nuclear associations. **A** total number of activity events recorded over 3 days. **B** the total number of activity events recorded when flies were not asleep. **C** The proportion of time that flies were determined to be asleep, defined as 5 min of inactivity, See Table 2 for outputs of statistical models.

See Fig. 3 and methods for model details. A full description of sequence polymorphism in these mtDNA variants, including synonymous, non-synonymous, tRNA SNPs and indels can been found in Table S2 in Salminen et al. (2017).

### Breaking up co-evolved mito-nuclear combinations affects activity and sleep patterns

As naturally occurring mito-nuclear genome combinations have co-adapted locally over time, we expected that disruption of these combinations could result in maladaptive effects. We also expected that these effects might be more severe, or more variable in males, as postulated under the mother’s curse hypothesis (Carnegie et al. 2021; Dowling and Adrian 2019). Both male and female cybrid flies were significantly more active than flies with co-evolved mito-nuclear combinations (on average, 75% more active), although the extent of this variation differed between sexes and was more prominent in females (Table 2 ‘Line × Sex’ effect; Fig. 3A). Part of the increase in total activity in cybrids was driven by a 57% increase in the awake activity level (2.2–3.8 recorded movements per 5-min bout, on average, in the original and cybrid line, respectively), though this was the case mainly in females (Table 2 ‘Line × Sex’ effect; Fig. 3B). However, the largest driver of the increased total activity in both male and female cybrid flies is that these spent a significantly lower proportion of their time asleep (38% of the time) compared to the co-evolved lines (59% of the time), and the extent of this difference was slightly larger in females (Table 2 ‘Line × Sex’ effect; Fig. 3C).

Given that our design was not orthogonal and only one nuclear genotype was tested across several mitochondrial genotypes, we cannot conclusively quantify the effect of nuclear variation. However, we are able to calculate the proportion of variance explained in each trait by mtDNA, as ratio of the among-line variance in the cybrids with the among-line variance in original lines (Table 4). This analysis shows that for both total activity and sleep, mtDNA explained only a fraction of variance within lines (total activity 20%; sleep 14%, Table 4). However, we were surprised to see that the among-line variance in awake activity in the original lines was very low (2%) and that when isolated on a common nuclear background, mtDNA explained six times more variance compared to the original lines (Table 4).

**Table 4:** Variance components.

	Total activity	Awake activity	Sleep
	variance	variance	variance
Original lines	5.79E + 05	0.02	0.48
Cybrid lines	1.14E + 05	0.15	0.07
Cybrid/Original ratio	0.20	6.04	0.14

The variance in each trait is explained by Line in the Original lines and in the Cybrid lines. In original lines, the variance originates from both the nuclear and mtDNA. In the cybrid lines, variance indicates the variance explained by the different mtDNAs once introgressed onto the ORT nuclear background. The ratio of Cybrid/Original variance indicated the proportion of total variance in each trait explained by mtDNA.

## Discussion

Mitochondrial dysfunction commonly manifests in tissues with high metabolic demands, such as muscles, with movement disorders being a major feature of mitochondrial diseases (Ghaoui and Sue 2018). However, studying the effect of mtDNA variation is challenging as it is necessary to disentangle the effects of the mitochondrial genome from the effect of the nuclear background. This is feasible with the *Drosophila* cybrid model, and here we have focused on studying the effect of mtDNA variation on locomotion activity and sleep. We addressed three questions by separately quantifying the contributions of the mitochondrial and nuclear genomes to activity and sleep phenotypes. First, we asked how variation in both the nDNA and mtDNA affects sleep and activity. Second, by isolating mtDNA variants on a common nuclear background we investigated how variation in mtDNA affects sleep and activity. Finally, we assessed how breaking up co-evolved mito-nuclear genetic interactions affected sleep and activity.

Locomotor activity and the proportion of time spent asleep were first measured from eight wild-type *Drosophila* strains with co-evolved mito-nuclear combination adapted to their local environment. These strains showed variation in their sleep-wake patterns and in general females were more active overall; males exhibited a higher waking activity but slept for a larger proportion of time. It is hypothesised that inseminated females spend more time awake as they have to lay eggs and scavenge for food to maintain high levels of fecundity. These results are supported by previous literature that finds genotype-specific and sexual dimorphism for this trait (Hyde and Jerussi 1983; Long and Rice 2007). We next aimed to test if mtDNA contributed to this variation between lines. The eight studied mtDNA genomes can be subdivided into two haplogroups based on their genetic variation (Salminen et al. 2017). Interestingly, haplogroup II females were shown to have higher total activity levels and spent less time sleeping when compared to haplogroup I females. The same was not observed with males. It is unclear how these patterns might have arisen in nature. However further experimentation, replicated in both lab and natural settings, is required to examine if these patterns are a result of local adaptation, or if they could arise due to independent compensatory mito-nuclear co-evolution, or even just due to drift.

As the eight cybrids also possess mitochondrial haplotype-specific mutations, we examined their effects on locomotion and sleep when introgressed into common nuclear background in the cybrid lines. In general, we were able to see differences in the amounts of activity and sleep, in both females and males, brought upon by mtDNA variation. Cybrid females were more active than males, sleep less and have higher waking activity. It is difficult to say if specific mtDNA mutations are causing the seen variation, as most of these mtDNA genomes contain more than one source of variation, i.e replacement variants in the protein-coding genes, synonymous SNPs, indels in the tRNA and rRNA genes and also length variation in the non-coding A + T region (Salminen et al. 2017). Here we focused on non-synonymous SNPs between our cybrid strains as they are predicted to have larger effect size, however several studies have shown that synonymous SNPs and SNPs in tRNAs also have a significant phenotypic effect (Meiklejohn et al. 2013; Camus et al. 2017). Mitovariant mtM2, the only variant that is originally from Australia, contains most unique non-synonymous mutations when compared to the other mitovariants (Table 1). mtM2 was also one of the lowest activity strains, and females especially spent more time sleeping when compared to the other cybrids. This might be due to altered interactions between mitochondrial and nuclear gene products (Pichaud et al. 2019). M2 flies which have the coevolved nuclear background actually appear to exhibit relatively high activity and low amount of sleep. Disruption of naturally occurring mito-nuclear combinations can result in interruption of precise interactions, leading to mito-nuclear incompatibilities, which have downstream deleterious fitness consequences (Mossman et al. 2019).

mtKSA2 is the only African variant, and it possesses two unique amino acid replacement variants in OXPHOS cIII (*CYTB*) and cIV (*COIII*) when compared to other cybrids (Salminen et al. 2017). mtKSA2 cybrids males exhibited the highest proportion of time spent sleeping. Also, the overall activity counts were among the lowest in mtKSA2 females and males, and the mtKSA2 cybrids females also had the lowest awake activity counts. The mtDNA variation studied here is maternally inherited and its effects can be multisystemic, affecting each tissue in Drosophila. However, there are cases where several sporadic missense and nonsense *CYTB* mutations in muscles have been shown to cause complex III deficiency and exercise intolerance in humans (Andreu et al. 1999). *CYTB* mutation in mtKSA2 may partially explain the lower activity rates when compared to other strains.

mtKSA2 is also associated with low mtDNA copy number in ORT nuclear background when compared to other cybrid lines (Salminen et al. 2017). Since mtDNA copy number has been shown to be associated with other measures of fitness in Drosophila including fertility and longevity (Camus et al. 2015) and with development time and weight (Salminen et al. 2017) of the same co-evolved and cybrid lines as studied here, it is possible that it might also be associated with variation in activity levels. mtDNA copy number is sexually dimorphic in Drosophila, with females of most strains tending to exhibit a higher copy number than males (Camus et al. 2015; Salminen et al. 2017), and is also affected by the age of the flies, sex specifically (Salminen et al. 2017). Sleep and activity are also shown to be sexually dimorphic traits in Drosophila (Long and Rice 2007; Shaw et al. 2000). In our study, we saw that in both co-evolved and cybrid strains the females were more active overall and slept less, as shown also with previous *Drosophila* sleep and activity research (Isaac et al. 2010). A future line of enquiry is therefore whether mtDNA copy number levels correlate with the extent of locomotor activity and sleep.

Previous studies have demonstrated that disrupting co-evolved mito-nuclear interactions can lead to decreased OXPHOS function (Sackton et al. 2003; Salminen et al. 2017). Specifically, cytochrome c oxidase activity has been shown to be reduced when an mtDNA haplotype is combined with a more distant nuclear background rather than its coevolved nuclear background in copepod *Tigriopus californicus* (Willett and Burton 2003). Mito-nuclear incompatibilities can lead to reduction in fitness (Clancy 2008; Clancy et al. 2011; Yee et al. 2013), and maintaining normal levels of activity is beneficial to fitness to allow, for example foraging and mating to take place (Long and Rice 2007). Short sleep duration in Drosophila has previously been shown to be associated with poor memory (Bushey et al. 2007), and reduced longevity (Bushey et al. 2010; Cirelli et al. 2005).

Natural selection is blind to deleterious mitochondrial phenotypes which manifest in males as mtDNA is maternally inherited (Carnegie et al. 2021; Frank 2012; Gemmell et al. 2004). This can indicate that variation which persists in mtDNA is more likely to cause greater phenotypic divergence within males and more likely to cause deleterious effects in males than in females (Frank 2012; Gemmell et al. 2004). While several studies provide evidence in support of this theory, known as the mother’s curse (Camus et al. 2012; Carnegie et al. 2021; Ruiz-Pesini et al. 2000), our data here do not suggest the mother’s curse impacts activity. Although we observed a general increase in activity and decrease in sleep in both males and female cybrids, the magnitude of these effects was larger in females, and not in males, as would be predicted under the mother’s curse hypothesis.

Drosophila sleep and activity research will enable further understanding of the causes of abnormal sleep and activity patterns in humans and the role of mitochondrial variation in these traits. Further studies of mito-nuclear interactions and mtDNA variation are essential in recognising and eventually preventing mito-nuclear mismatches that might occur during mitochondrial replacement therapy.

### Data archiving

All datasets and full R code for all analyses can be found at 10.5281/zenodo.5573904 and is fully citable as Vale PF (2021) Data and code for Mitonuclear interactions affect locomotor activity and sleep in Drosophila melanogaster [Data set]. Zenodo. 10.5281/zenodo.5573904.

## Supplementary information

Supplementary Figures and tables
