# Supplementary material for: Variation in mitochondrial DNA affects locomotor activity and sleep in Drosophila melanogaster
Source: Heredity (Edinb). 2022 Jun 28;129(4):225–32. doi: 10.1038/s41437-022-00554-w (PMC9519576; doi:10.1038/s41437-022-00554-w)
Supplement: Supplementary file 1 — Supplementary Figures and tables [file 41437_2022_554_MOESM1_ESM.pdf]

Supplementary Figures and tables

Figure S1 – Actograms for original lines with coevolved nuclear and mitochondrial genomes

Figure S2. **Variation in activity and sleep in original lines**. Locomotor activity and sleep in females (red) and males (blue) of the coevolved fly lines. **A)** total number of activity events recorded over three days. **B)** the total number of activity events recorded when the flies were awake. **C)** The proportion of time that flies were determined to be asleep, defined as 5 min of inactivity, See Fig S1 for individual actograms and Table 1 for details of each line. See Table 2 for outputs of statistical models.

Figure S3– Actograms for cybird lines with novel nuclear and mitochondrial combinations

Figure S4. Mitochondrial haplotype effect. Locomotor activity and sleep in cybrid females and males. Each mtDNA variant was introgressed onto the ORT nuclear background (ORT also included here to allow direct comparisons). **A**) total number of activity events recorded over three days. **B**) the total number of activity events recorded when flies were not asleep. **C**) The proportion of time that flies were determined to be asleep, defined as 5 min of inactivity, See Fig S2 for individual actograms and Table 1 for details of each mitochondrial haplotype. See Table 2 for outputs of statistical models.

Table S1 – Variance components for the random effects

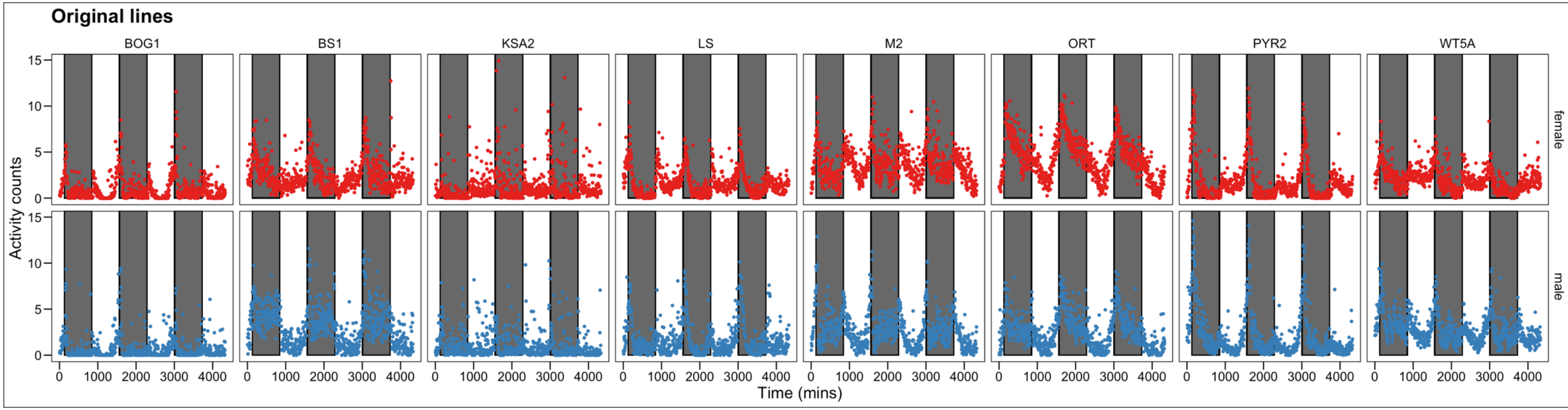

Fig S1 – Drosophila Activity Monitor (DAM) Activity counts (unique breaks of the infra-red beam) measured for each original fly line over a period of three days. Females are shown in the top panel in red; males in the bottom panel in blue. Within each plot, the white vertical bars indicate periods of light, while the dark vertical bars indicate periods of dark when lights are turned off within the incubator. Details about each line can be found in Table 1. Summary statistics for the activity patters can be found in Figure 1.

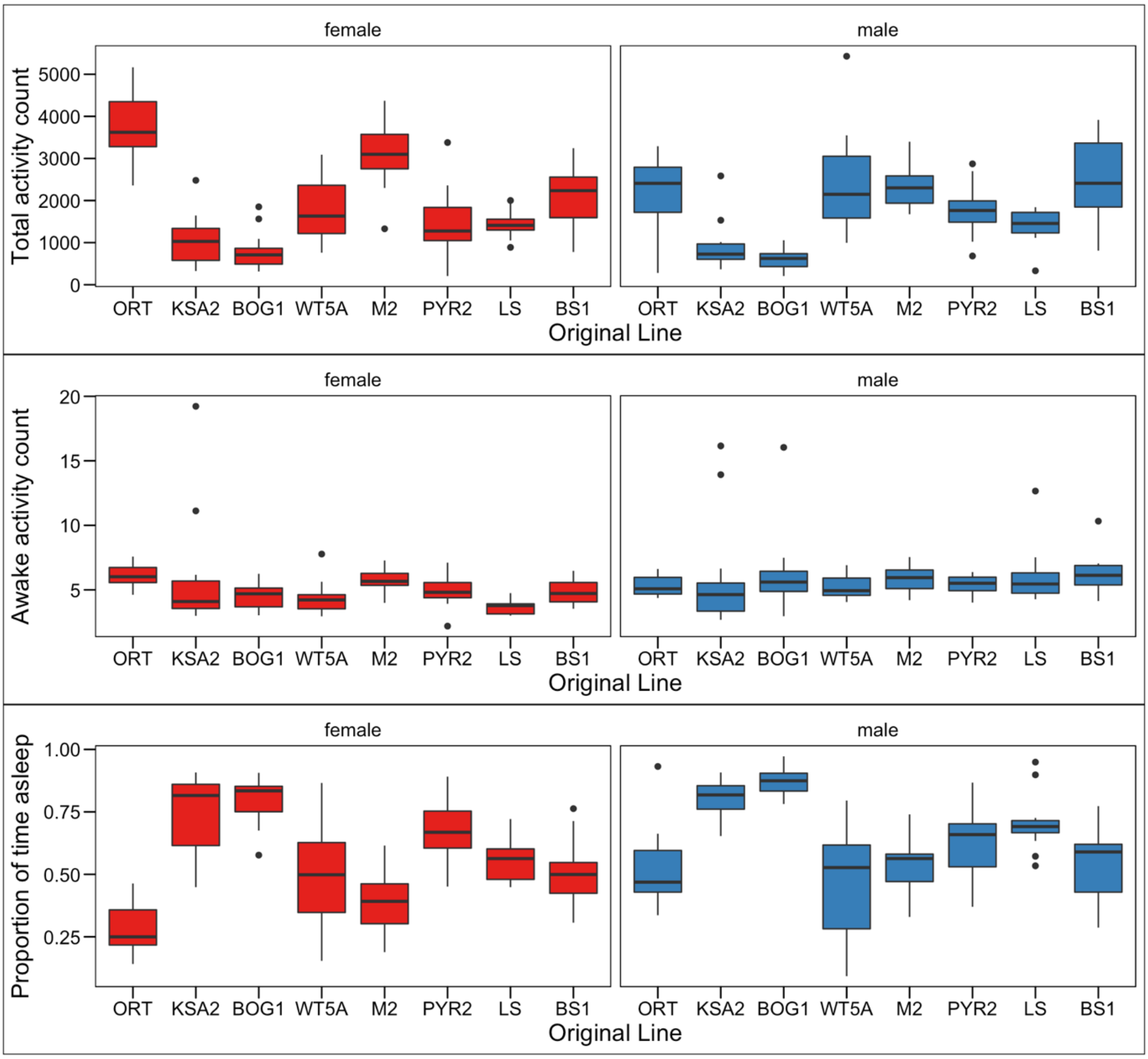

Figure S2. **Variation in activity and sleep in original lines**. Locomotor activity and sleep in females (red) and males (blue) of the coevolved fly lines. **A)** total number of activity events recorded over three days. **B)** the total number of activity events recorded when the flies were awake. **C)** The proportion of time that flies were determined to be asleep, defined as 5 min of inactivity, See Fig S1 for individual actograms and Table 1 for details of each line. See Table 2 for outputs of statistical models.

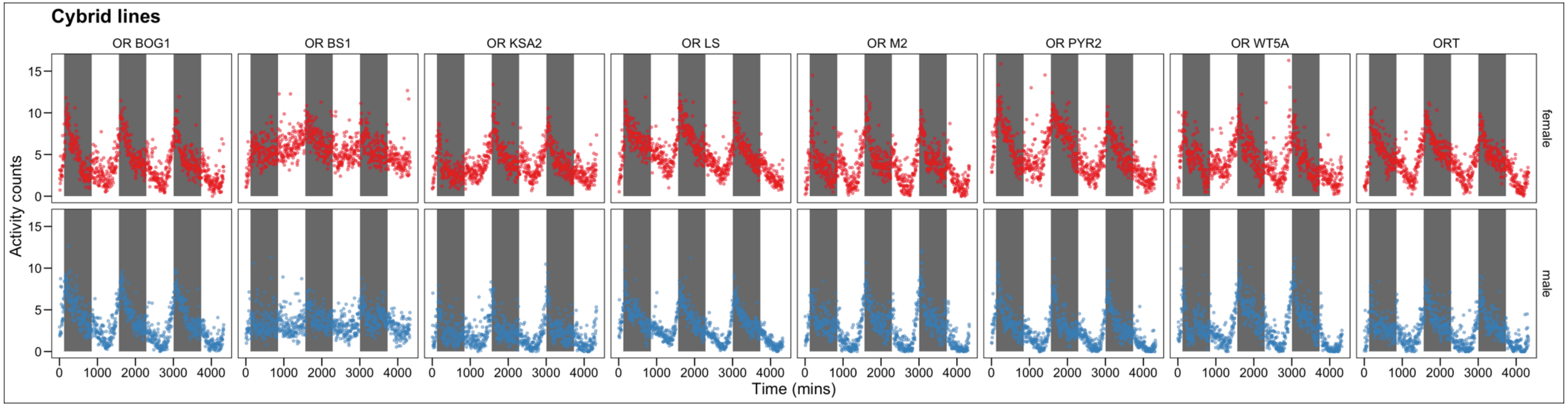

Fig S3 - Drosophila Activity Monitor (DAM) Activity counts over a period of three days (unique breaks of the infra-red beam) measured for each cybrid fly line, where each mitochondrial haplotype has been introgressed onto the OR nuclear background. Females are shown in the top panel in red; males in the bottom panel in blue. Within each plot, the white vertical bars indicate periods of light, while the dark vertical bars indicate periods of dark when lights are turned off within the incubator. Details about each line can be found in Table 1. Summary statistics for the activity patters can be found in Figure 2.

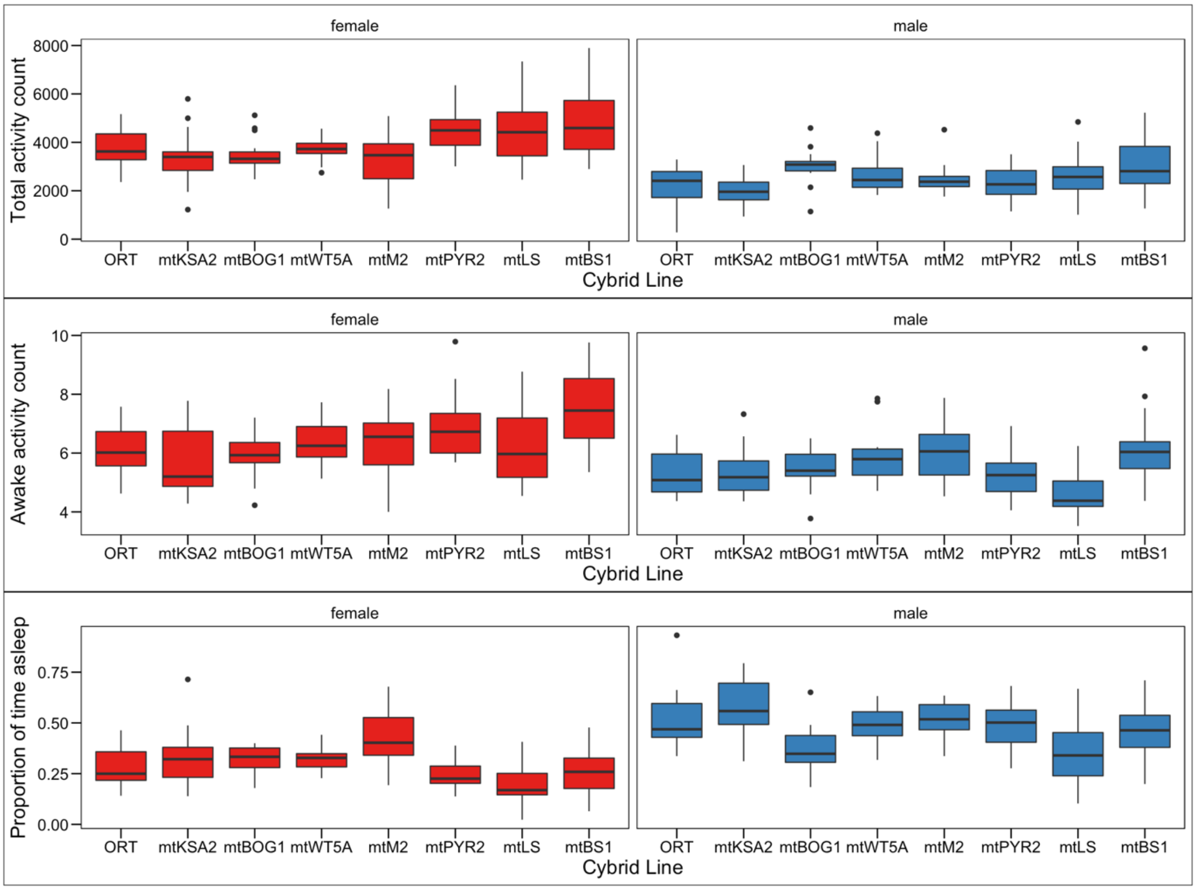

Figure S4. Mitochondrial haplotype effect. Locomotor activity and sleep in cybrid females and males. Each mtDNA variant was introgressed onto the ORT nuclear background (ORT also included here to allow direct comparisons). **A**) total number of activity events recorded over three days. **B**) the total number of activity events recorded when flies were not asleep. **C**) The proportion of time that flies were determined to be asleep, defined as 5 min of inactivity, See Fig S2 for individual actograms and Table 1 for details of each mitochondrial haplotype. See Table 2 for outputs of statistical models.

Original lines	Total activity		Awake activity		Sleep	
Random effects	**variance**	**Std. Dev.**	**variance**	**Std. Dev.**	**variance**	**Std. Dev.**
Replicate	0	0	7.00E-10	2.65E-05	0.022	0.15
Block / Run	5046	71	0.03	0.16	0.027	0.16
Residual	437448	661	3.40	1.84		

Cybrid lines	Total activity		Awake activity		Sleep	
Random effects	**variance**	**Std. Dev.**	**variance**	**Std. Dev.**	**variance**	**Std. Dev.**
Replicate	0	0	1.54E-03	0.04	0.122	0.35
Block / Run	5884	76.71	0.03	0.16	0.202	0.45
Residual	8.50E+05	922.08	0.92	0.96		

Table S1. Random effect variance and standard deviation. See Table 2 for fixed effects.
